# Photosynthetic flexibility in maize exposed to salinity and shade

**DOI:** 10.1093/jxb/eru130

**Published:** 2014-04-01

**Authors:** Robert E. Sharwood, Balasaheb V. Sonawane, Oula Ghannoum

**Affiliations:** Hawkesbury Institute for the Environment, University of Western Sydney, Richmond, Locked bag 1797, Penrith NSW 2751, Australia

**Keywords:** C4 photosynthesis, carbon isotope discrimination, leakiness, NADP-ME, PEP-CK, PEPC, Rubisco.

## Abstract

C_4_ photosynthesis involves a close collaboration of the C_3_ and C_4_ metabolic cycles across the mesophyll and bundle-sheath cells. This study investigated the coordination of C_4_ photosynthesis in maize plants subjected to two salinity (50 and 100mM NaCl) treatments and one shade (20% of full sunlight) treatment. Photosynthetic efficiency was probed by combining leaf gas-exchange measurements with carbon isotope discrimination and assaying the key carboxylases [ribulose-1,5-bisphosphate carboxylase/oxygenase (Rubisco) and phosphoenolpyruvate carboxylase (PEPC)] and decarboxylases [nicotinamide adenine dinucleotide phosphate malic enzyme (NADP-ME) and phosphoenolpyruvate carboxykinase (PEP-CK)] operating in maize leaves. Generally, salinity inhibited plant growth and photosynthesis to a lesser extent than shade. Salinity reduced photosynthesis primarily by reducing stomatal conductance and secondarily by equally reducing Rubisco and PEPC activities; the decarboxylases were inhibited more than the carboxylases. Salinity increased photosynthetic carbon isotope discrimination (Δ_p_) and reduced leaf dry-matter carbon isotope composition (^13^δ) due to changes in *p*_i_/*p*_a_ (intercellular to ambient CO_2_ partial pressure), while CO_2_ leakiness out of the bundle sheath (ϕ) was similar to that in control plants. Acclimation to shade was underpinned by a greater downregulation of PEPC relative to Rubisco activity, and a lesser inhibition of NADP-ME (primary decarboxylase) relative to PEP-CK (secondary decarboxylase). Shade reduced Δ_p_ and ɸ without significantly affecting leaf ^13^δ or *p*_i_/*p*_a_ relative to control plants. Accordingly, shade perturbed the balance between the C_3_ and C_4_ cycles during photosynthesis in maize, and demonstrated the flexible partitioning of C_4_ acid decarboxylation activity between NADP-ME and PEP-CK in response to the environment. This study highlights the need to improve our understanding of the links between leaf ^13^δ and photosynthetic Δ_p_, and the role of the secondary decarboxylase PEP-CK in NADP-ME plants such as maize.

## Summary

In maize, salinity reduced photosynthesis by decreasing stomatal conductance. Shade downregulated PEPC more than Rubisco, leading to decreased bundle-sheath CO_2_ leakiness. The treatments elicited plasticity in NADP-ME/PEP-CK activity ratio.

## Introduction

C_4_ photosynthesis evolved as a spatial and biochemical adaptation to remedy the inefficiency of C_3_ photosynthesis under conditions of high temperature, low CO_2_, and water stress, all of which exacerbate photorespiration (Ludwig, 2013). The propensity of photorespiration is determined by the extent of oxygenation carried out by ribulose-1,5-bisphosphate carboxylase/oxygenase (Rubisco) (Andrews *et al.*, 1973; Cleland *et al.*, 1998). For C_3_ plants, the current O_2_ concentration in the atmosphere (210 mmol mol^–1^) severely inhibits the carboxylation of ribulose-1,5-bisphosphate by Rubisco. Together, these environmental conditions are hypothesized to be the drivers for the independent evolution of plants operating a CO_2_ concentration mechanism (CCM; Sage *et al.*, 2012). The distinguishing features of the CCM in most C_4_ plants include the operation of two metabolic cycles (C_3_ and C_4_) across two photosynthetic cell types, mesophyll cells (MCs) and bundle-sheath cells (BSCs), which compartmentalize the initial carboxylation and decarboxylation reactions (Hatch, 1987; Langdale, 2011). The primary step of HCO_3_^–^ fixation to phosphoenolpyruvate is catalysed by phosphoenolpyruvate carboxylase (PEPC) to produce oxaloacetate, which is subsequently converted into C_4_ acids within the MCs (Jenkins *et al.*, 1987). These organic acids then diffuse into neighbouring BSCs where decarboxylation of C_4_ acids releases CO_2_. A high CO_2_ concentration within the semi-gas-tight BSCs suppresses photorespiration and enhances the capacity for CO_2_ fixation by Rubisco. C_4_ photosynthesis has three biochemical subtypes depending on the C_4_ decarboxylase enzyme: nicotinamide adenine dinucleotide phosphate malic enzyme (NADP-ME), NAD malic enzyme, and phosphoenolpyruvate carboxykinase (PEP-CK) (Gutierrez *et al.*, 1974; Kanai and Edwards, 1999). C_4_ plants have been classified into one of the three subtypes based on the dominant C_4_ acid decarboxylation enzyme. Specialized leaf anatomy, biochemistry, and physiology are associated with each of the C_4_ subtypes (Ghannoum *et al.*, 2005, 2011).

Nevertheless, there is evidence emerging that PEP-CK activity is more widespread among the biochemical subtypes, suggesting that a degree of flexibility within the C_4_ cycle may exist depending on species or environmental conditions (Leegood and Walker, 2003; Furbank, 2011). In maize, an NADP-ME C_4_ grass, 25% of the oxaloacetate produced is cycled through an alternative pathway involving the aspartate aminotransferase shuttle and the subsequent decarboxylation of oxaloacetate within the cytosol of BSCs catalysed by PEP-CK (Wingler *et al.*, 1999; Furbank, 2011). This has been shown to exist for maize and other C_4_ grasses (Gutierrez *et al.*, 1974). Therefore, the presence of alternative decarboxylase pathways within maize provides the possibility for flexibility in the use of the decarboxylation pathways of the CCM under certain growth conditions (Leegood and Walker, 2003).

For C_4_ plants, there is an additional energetic cost associated with the operation and overcycling of the CCM. Minimally, an extra two ATP molecules per CO_2_ fixed are required for the regeneration of PEP from pyruvate. During C_4_ photosynthesis, the C_4_ cycle operates faster than the C_3_ (Calvin) cycle in order to raise the BSC CO_2_ concentration and saturate the carboxylation reaction of Rubisco. Inevitably, a fraction of this CO_2_ is not fixed by Rubisco and ultimately leaks back from the BSCs to the MCs. This fraction is termed leakiness (ɸ) and entails additional energy costs associated with the overcycling of the C_4_ cycle (Farquhar, 1983; Furbank *et al.*, 1990). Consequently, the efficiency of C_4_ photosynthesis requires the tight regulation of CO_2_ supply with Rubisco activity within the BSCs in order to minimize leakiness and associated energy costs. This is often the case, given that leakiness varies within a narrow range and averages about 20% for a wide range of C_4_ plants and environments (Henderson *et al.*, 1992; von Caemmerer *et al.*, 1997*a*; Cousins *et al.*, 2008). Bundle-sheath leakiness can be estimated by concurrently measuring leaf gas exchange with carbon isotope discrimination (Evans *et al.*, 1986). A number of studies have examined the effects of short-term and long-term changes in environmental parameters, such as light, water stress, and salinity, yielding mixed results. A few studies have estimated leakiness from measurements of dry-matter carbon isotope, and found that leakiness was impacted by light, salinity, or water stress (Buchmann *et al.*, 1996; Saliendra *et al.*, 1996; Fravolinil *et al.*, 2002). When leakiness was estimated from carbon isotope discrimination measured during gas exchange, small changes in leakiness have been reported in some studies but not others in response to short- or long-term changes in the environment (Bowman *et al.*, 1989; Kubasek *et al.*, 2007). In particular, Bowman *et al.* (1989) found that leakiness changed diurnally in salt-stressed *Zea mays* and *Andropogon glomeratus*, two C_4_, NADP-ME grasses, while Kubasek *et al.* (2007) reported that leakiness increased with low light and low temperature. Lowering light intensity during gas-exchange measurements had no effect on bundle-sheath leakiness in a number of C_4_ plants (Henderson *et al.*, 1992), and leakiness was unchanged under long-term exposure to low light (Bellasio and Griffiths, 2013*a*). Ubierna *et al.* (2013) found that the increase in leakiness commonly reported at low light (Henderson *et al.*, 1992) was only marginally present when using the full model for carbon isotope discrimination in C_4_ leaves (Farquhar and Cernusak, 2012). Leakiness depends on a number of anatomical (e.g. CO_2_ diffusion path length, chloroplast position in the BSC, BSC wall conductance) and biochemical (e.g. activities of the carboxylases and decarboxylases during C_4_ photosynthesis) factors (Henderson *et al.*, 1992; von Caemmerer and Furbank, 2003). In contrast to manipulations using transgenic C_4_ plants (von Caemmerer *et al.*, 1997*b*; Cousins *et al.*, 2006; Pengelly *et al.*, 2012), few studies have investigated the effects of environmental variables on leakiness together with possible underlying biochemical mechanisms.

Consequently, the current study was aimed at investigating the efficiency of C_4_ photosynthesis in maize exposed to long-term shade and salinity, by combining measurements of leakiness with assays of the two carboxylases and decarboxylases known to operate in maize leaves. A second aim of this study was to probe the plasticity of the C_4_ acid decarboxylases in response to these environmental variables. Salinity and shade were chosen because they impact on photosynthesis through contrasting effects on leaf CO_2_ diffusion and fixation. Mild to moderate salinity inhibits root water uptake, thus indirectly reducing the plant water status, as detected by increased leaf water potential and reduced stomatal conductance, both of which reduce photosynthesis (Munns and Tester, 2008; Omoto *et al.*, 2012; Shabala and Munns, 2012). Low light reduces photosynthesis mainly by reducing activity and activation of photosynthetic enzymes (Edwards *et al.*, 1985).

## Materials and methods

### Plant culture

Maize seeds (Sweet Corn, Kelvedon Glory 5713) were germinated in 5 l pots (shaded plants were raised in 2 l pots) containing standard potting mix in a sunlit glasshouse during summer (December–March 2012). Nutrients were supplied through the addition of Osmocote and periodic watering with soluble Aquasol supplemented with magnesium sulfate. Maize plants destined for the salinity treatments were initially watered with tap water. Once seedlings were well established (2 weeks after germination), NaCl was added at increasing concentrations to the watering solution over a period of 2 weeks until the endpoint concentrations of 50 and 100mM NaCl were reached. To minimize NaCl accumulation, pots were flushed with water once a week, and then irrigated with the desired NaCl concentration. Plants destined for shading were germinated as above in full sunlight and then placed under a shade cloth, which limited light to 20% of the ambient sunlight. At midday, the photosynthetic active radiation of full sunlight ranged between 1000 and 1800 µmol m^–2^ s^–1^ when measured at pot level during the experiment. Air temperature inside the glasshouse compartment was regulated by a temperature-control system, and day/night temperatures averaged 26/18 °C. Relative humidity was monitored and ranged between 60 and 80% during the day. There were five pots per treatments. Plants were harvested 12 weeks after germination.

### Measuring leaf gas exchange

Leaf gas-exchange measurements were carried out 1–2 weeks before harvest using a portable open photosynthesis system (LI-6400XT; LI-COR, Lincoln, USA). Measurements of light-saturated photosynthetic rate (*A*_sat_) and stomatal conductance (*g*_s_) were taken between 10:00 and 14:00 at ambient CO_2_ (400 µl l^–1^), a leaf temperature of 26 °C, and a photosynthetic photon flux density of 1800 µmol m^–2^ s^–1^. Each leaf was allowed to reach a steady state of CO_2_ uptake in the LI-6400XT leaf chamber before measurements were taken.

Photosynthetic responses to intercellular CO_2_ concentration (*A*/*C*_i_ curves) were measured at 10 CO_2_ steps using similar conditions as described above. The *A*/*C*_i_ curves were fitted using a C_4_ photosynthesis model (von Caemmerer, 2000) to estimate maximal PEPC (*in vivo V*_pmax_) and Rubisco (*in vivo V*_cmax_) activities. *V*_cmax_ and *V*_pmax_ were varied simultaneously until the best fit with the gas-exchange data was obtained.

### Photosynthetic carbon isotope discrimination

Bundle-sheath leakiness was determined by measuring real-time ^13^CO_2_/^12^CO_2_ carbon isotope discrimination using a gas exchange system (LI-6400XT: LI-COR) attached to a tunable diode-laser (model TGA100; Campbell Scientific, Logan, UT, USA), under similar conditions to the spot gas exchange measurements. Photosynthetic discrimination against ^13^C (Δ_p_) was calculated using the following equations (Evans *et al.*, 1986):

$$\Delta_{p}=\frac{\xi (\delta_{\text{o}}-\delta_{\text{e}})}{1+\delta_{\text{o}}-\xi \left(\delta_{\text{o}}-\delta_{\text{e}}\right)}$$ (1)

$$\xi =\;\frac{C_{\text{e}}}{C_{\text{e}}-C_{\text{o}}}$$ (2)

where *δ*_*e*_, *δ*_*o*_, *C*_e_, and *C*_o_ are the δ^13^C (δ) and CO_2_ mol fraction (*C*) of the air entering (e) and leaving (o) the leaf chamber and were measured with the tunable diode-laser. In this study, ξ ranged between 5 and 11. Leakiness (ɸ) was calculated using the model of Farquhar (1983) as modified by Pengelly *et al*. (2010, 2012). The formulae used are described briefly below.

$$\phi =\;\frac{\left(\frac{1-t}{1+t}\right)\Delta -\frac{a\prime }{1+t\;}-(a_{i}-b_{4}^{\prime })\frac{A}{g_{\text{m}}C_{\text{a}}}-\;\left(b_{4}^{\prime }-\;a^{\prime }\right)\frac{C_{\text{i}}}{C_{\text{a}}}}{(b_{3}^{\prime }-s)\left(\frac{C_{\text{i}}}{C_{\text{a}}}-\frac{A}{C_{\text{a}}g_{\text{m}}}\right)}$$ (3)

where the term *t*, which represents the ternary effect, is defined as by Farquhar and Cernusak, (2012):

$$t=\;\frac{(1+a^{\prime })E}{2g_{\text{ac}}^{\text{t}}}$$ (4)

where *E* is the transpiration rate and *g* ^t^_ac_ the total conductance to CO_2_ diffusion including boundary layer and stomatal conductance (von Caemmerer and Farquhar, 1981). The symbol *a*′ denotes the combined fractionation factor through the leaf boundary layer and through stomata:

$$a^{\prime }=\frac{a_{\text{b}}\left(C_{\text{a}}-\;C_{\text{ls}}\right)+a\left(C_{\text{ls}}-\;C_{\text{i}}\right)}{C_{\text{a}}-\;C_{\text{i}}}$$ (5)

where *C*_a_, *C*_i_, and *C*_ls_ are the ambient, intercellular, and leaf surface CO_2_ partial pressures, *a*_b_ (2.9‰) is the fractionation occurring through diffusion in the boundary layer, *a* (4.4‰) is the fractionation due to diffusion in air (Evans *et al.*, 1986), *s* (1.8‰) is the fractionation during leakage of CO_2_ out of the bundle sheath, and *a*_i_ is the fractionation factor associated with the dissolution of CO_2_ and diffusion through water. Here, we assume that *s=a*_i_.

$$b_{3}^{\prime }=b_{3}-e\left(\frac{R_{\text{d}}}{A+R_{\text{d}}}-\frac{0.5R_{\text{d}}}{A+0.5R_{\text{d}}}\right)-f\frac{0.5V_{0}}{V_{\text{c}}}$$ (6)

and

$$b_{4}^{\prime }=b_{4}-\;e\frac{0.5\;R_{\text{d}}}{\left(A+0.5R_{\text{d}}\right)}$$ (7)

where *b*_3_ is the fractionation by Rubisco (30‰), *b*_4_ is the combined fractionation of the conversion of CO_2_ to HCO_3_^−^ and PEP carboxylation (–5.74‰ at 25 °C), *f* is the fraction associated with photosrespiration, and *V*_o_ and *V*_*c*_ are the rates of oxygenation and carboxylation, respectively. The fractionation factor *e* associated with respiration was calculated from the difference between δ^13^C in the CO_2_ cylinder (–40.5‰) used during experiments and that in the atmosphere under growth conditions (–8‰; Tazoe *et al.*, 2008). *A* and *R*_d_ denote the CO_2_ assimilation rate and day respiration, respectively; *R*_d_ was assumed to equal dark respiration. We assumed a mesophyll conductance (*g*_m_)=1mol m^−2^ s^−1^ bar^−1^ for these calculations. In this study, leaf gas exchange was measured at high light, and hence *V*_o_=0 ($\text{i}.\text{e}.f\frac{0.5V_{0}}{V_{c}}$ =0) (Pengelly *et al*., 2010, 2012; Ubierna *et al.*, 2013).

### Rubisco content and soluble protein determination

Following gas-exchange measurements, replicate leaf discs (0.74cm^2^) were rapidly frozen in liquid nitrogen and then stored at –80 °C until analysed. Each leaf disc was extracted in 1ml of ice-cold extraction buffer [50mM EPPS/NaOH (pH 8.0), 5mM dithiothreitol, 20mM NaHCO_3_, 20mM MgCl_2_, 1mM EDTA, 4% (v/v) Protease Inhibitor Cocktail (Sigma), and 1% (w/v) polyvinyl polypyrrolidone] using a 2ml Potter–Elvehjem glass homogenizer kept on ice. Subsamples were taken from the total extract for SDS-PAGE analysis (see below) of total leaf protein. The remaining extract was centrifuged at 16, 100*g* for 1min and the supernatant used for Rubisco and soluble protein assays. Rubisco content was estimated by the irreversible binding of [^14^C]carboxyarabinitol bisphosphate (CABP) to the fully carbamylated enzyme (Sharwood *et al.*, 2008). Extractable soluble proteins were measured using a Coomassie Plus kit (Pierce).

### Activity of carboxylase and decarboxylase enzymes

Activity of Rubisco in maize extracts was determined by multiplying the number of Rubisco active sites determined using the [^14^C]CABP binding assay by the Rubisco *in vitro k*_cat_ (5.5 s^–1^) determined using a ^14^CO_2_ fixation assay (Sharwood *et al.*, 2008). The activity of the PEPC and NADP-ME enzymes were determined spectrophotometrically as described previously (Ashton *et al.*, 1990; Pengelly *et al.*, 2012).

The activity of PEP-CK in maize extracts was measured in the carboxylase direction using the method outlined by Walker *et al.* (2002). For each assay, a separate leaf disc was homogenized in extraction buffer containing 50mM HEPES (pH 7.2), 5mM dithiothreitol, 1% polyvinyl polypyrrolidone, 2mM EDTA, 2mM MnCl_2_, and 0.05% Triton X-100. MgCl_2_ was not added to the extraction or assay buffer to remove the possibility of interference from other enzymes. PEP-CK activity was measured in assay buffer [100mM HEPES (pH 7.0), 4% mercaptoethanol (w/v), 100mM KCl, 90mM NaHCO_3_, 1mM ADP, 2mM MnCl_2_, 0.14mM NADH, and malate dehydrogenase (6U)] after the addition of PEP to 5mM. The final concentration of 4mM MnCl_2_ has been shown to be sufficient for PEP-CK activity (Chen *et al.*, 2002; Walker *et al.*, 2002).

### SDS-PAGE and immunoblot analysis of Rubisco and CCM proteins

Subsamples of total protein fractions were mixed with 0.25 vols of 4× LDS buffer (Invitrogen) containing 100mM dithiothreitol and placed on ice until analysed within 2h. For confirmatory visualization, protein samples were separated by SDS-PAGE in TGX Any kD (BioRad) pre-cast polyacrylamide gels buffered with 1× Tris/glycine SDS buffer (BioRad) at 200V using a Mini-Protean apparatus at 4 °C. Proteins were visualized by staining with Bio-Safe Coomassie G-250 (BioRad) and imaged using a VersaDoc imaging system (BioRad).

For immunoblot analyses of total leaf protein, samples were separated by SDS-PAGE as outlined above and then transferred at 4 °C to nitrocellulose membranes (0.45 µm; BioRad) using a Xcell Surelock western transfer module (Invitrogen) buffered with 1× transfer buffer [20×: 25mM Bicine, 25mM Bis/Tris, 1mM EDTA, 20% (v/v) methanol]. After 1h transfer at 30V, the membrane was placed in blocking solution [3% (w/v) skimmed milk powder in TBS, 50mM Tris/HCl (pH 8), 150mM NaCl)] for 1h at room temperature with gentle agitation.

Primary antisera raised in rabbit against tobacco Rubisco (prepared by S. M. Whitney, Australian National University, Canberra, Australia) was diluted 1:4000 in TBS before incubation at 1h with membranes at room temperature with gentle agitation. Antisera raised against PEPC was obtained from AgriSera and diluted 1:2000 with TBS. For NADP-ME and PEP-CK, synthetic peptides based on monocot amino acid sequences for each protein were synthesized by GL Biochem and antisera were raised against each peptide in rabbits. The reactive antisera were the antigen purified for use in immunoblot analysis (GL Biochem). The NADP-ME and PEP-CK antisera were diluted in TBS at 1:1000 and 1:500, respectively.

All primary antisera were incubated with membranes at room temperature for 1h with gentle agitation before washing three times with TBS. Secondary goat anti-rabbit antiserum conjugated to horseradish peroxidase (Perkin Elmer) was diluted 1:3000 in TBS and incubated with the membranes for 1h at room temperature followed by three washes with TBS. Immunoreactive peptides were detected using an Immun-Star WesternC kit (BioRad) and imaged using VersaDoc.

### Plant biomass, leaf water potential, and nitrogen and carbon isotope composition

Before harvest, leaf water potential (Ψ_L_) was measured on a cut, matching gas-exchange leaf using a Scholander-style pressure chamber (PMS Instrument Company, Corvallis, OR, USA). At harvest, leaves were sampled and their area determined using a leaf area meter (LI-3100A; LI-COR) and roots were washed free of soil. Plant tissues were oven dried at 80 °C for 48h, weighed, and ground to a homogenous powder in a ball mill (MM-400; Retsch).

Leaf N content was determined on the ground tissue using a CN analyser (LECO TruSpec; LECO Corp., MI, USA). For carbon isotope composition (^13^δ), ground leaf samples were combusted in a Carlo Erba elemental analyser (Model 1108) and the CO_2_ was analysed by mass spectrometry. Isotopic composition (δ) was calculated as [(*R*_sample_ – *R*_standard_)/*R*_standard_]×1000, where *R*_sample_ and *R*_standard_ are the ^13^C/^12^C ratios of the sample and standard (Pee Dee Belemnite), respectively.

### Statistical analysis

Statistical significance tests were conducted using one-way analysis of variance computed in a general linear model. Treatment means were ranked using a post-hoc Tukey test.

## Results

### Plant growth and leaf nitrogen

**Fig. 1.**
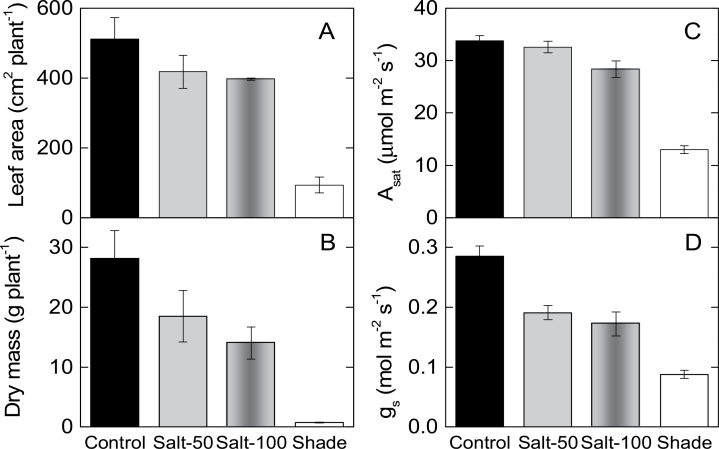
Growth of maize plants exposed to salinity and shade. Total leaf area (A), plant dry mass (B), light-saturated photosynthetic rates in ambient air, *A*_sat_ (C), and stomatal conductance, *g*_s_ (D), of maize plants grown in full sunlight and irrigated with water (control), 50mM NaCl (Salt-50), or 100mM NaCl (Salt-100), or grown in 20% sunlight (shade).

Plant leaf area was reduced by 18 and 22% for plants exposed to 50 and 100mM NaCl, respectively, whereas plant biomass was decreased by 34 and 50% for the same treatments when compared with the control (Fig. 1A, B, Table 1). Leaf mass per area was not significantly affected by salinity (Table 1).

**Table 1.** Summary of plant growth, leaf chemistry, leaf gas exchange and photosynthetic enzyme activity determined for maize plants grown in full sunlight and irrigated with normal water (control), 50mM NaCl (Salt-50) or 100mM NaCl (Salt-100) Shade plants were grown in 20% sunlight and irrigated with normal water.Values are treatment means of three replicates±standard error. Statistical significance tests were conducted using one-way analysis of variance computed in a general linear model. Treatment means were ranked using a post-hoc Tukey test, and values followed by the same letter are not significantly different at the 5% level (*P*<0.05). ND, not determined

Parameter	Control	Shade	Salt-50	Salt-100	Model *P value*
**Plant and leaf traits**					
Total leaf area (cm^2^ plant^–1^)	512±61*b*	94±23*a*	418±47*b*	397±4*b*	0.0012
Plant dry mass (g plant^–1^)	28.2±4.6*c*	0.75±0.05*a*	18.5±4.3*bc*	14.0±2.7*b*	0.0031
Leaf mass per area (g m^–2^)	71±5*b*	26±2*a*	69±6*b*	59±15*b*	0.0343
Leaf water potential, Ψ_L_ (MPa)	0.32±0.07*a*	ND	0.53±0.10*a*	1.08±0.09*b*	0.0000
Leaf N content (mg g^–1^)	36±2*b*	38±0*b*	33±2*ab*	29±1*a*	0.0054
Leaf N content (mmol m^–2^)	184±10*a*	95±25*a*	132±24*a*	124±28*a*	0.1027
Leaf C isotope composition, ^13^δ (‰)	–14.68±0.24*a*	–14.38±0.19*a*	–15.43±0.05*b*	–15.85±0.07*b*	0.0006
**Leaf gas exchange**					
Photosynthesis, *A*_sat_ (µmol m^–2^ s^–1^)	33.7±1.0*c*	13.0±0.7*a*	32.6±1.1*c*	28.3±1.6*b*	0.0000
Stomatal conductance, *g*_s_ (mol m^–2^ s^–1^)	0.285±0.017*c*	0.088±0.007*a*	0.191±0.012*b*	0.172±0.020*b*	0.0000
*in vivo V*_cmax_ (µmol m^–2^ s^–1^)	40±10*b*	19±6*a*	40±1*b*	33±1*b*	0.0125
*in vivo V*_pmax_ (µmol m^–2^ s^–1^)	104±6*b*	45±5*a*	94±1*b*	94±1*b*	0.0012
*V*_pmax_/*V*_cmax_	2.6±0.2*a*	2.5±0.4*a*	2.3±0.1*a*	2.8±0.1*a*	0.4682
Photosynthetic Δ_p_ (‰)	3.66±0.13*b*	2.46±0.36*a*	3.90±0.04*b*	4.23±0.11*b*	0.0040
Leakiness, ɸ	0.26±0.02*ab*	0.13±0.04*a*	0.24±0.02*ab*	0.31±0.01*b*	0.0125
**Photosynthetic enzymes**					
Rubisco content (g m^–2^)	0.31±0.04*b*	0.14±0.03*a*	0.23±0.01*ab*	0.24±0.03*ab*	0.0141
Soluble proteins (g m^–2^)	4.2±0.2*b*	2.5±0.2*a*	3.2±0.2*ab*	3.9±0.4*b*	0.0057
Rubisco activity (µmol m^–2^ s^–1^)	27.5±1.4*c*	11.5±2.0*a*	18.1±0.8*b*	19.5±2.1*b*	0.0034
PEPC activity (µmol m^–2^ s^–1^)	107±7*d*	21±4*a*	52±4*b*	72±4*c*	0.0000
PEPC/Rubisco	3.9±0.10*c*	1.8±0.08*a*	2.9±0.13*b*	3.3±0.15*b*	0.0000
NADP-ME activity (µmol m^–2^ s^–1^)	53±8*b*	32±5*a*	18±0.1*a*	19±3*a*	0.0073
PEP-CK activity (µmol m^–2^ s^–1^)	12.4±1.6*c*	3.0±0.4*a*	7.6±0.7*b*	8.2±1.2*b*	0.0021

The impact of 80% shading (shaded plants received 20% of ambient sunlight) on the maize plants was profound. Leaf area and total plant biomass were reduced to 18 and 3% of that of the control plants, respectively, while leaf mass per area was reduced to 37% of that of the control plants (Fig. 1A, B, Table 1).

Leaf N content per unit mass decreased in the high-salt-treated plants only relative to the control. When expressed on an areas basis, leaf N concentration tended to be lower in the shaded plants relative to the control (Table 1).

### Leaf photosynthesis

**Fig. 2.**
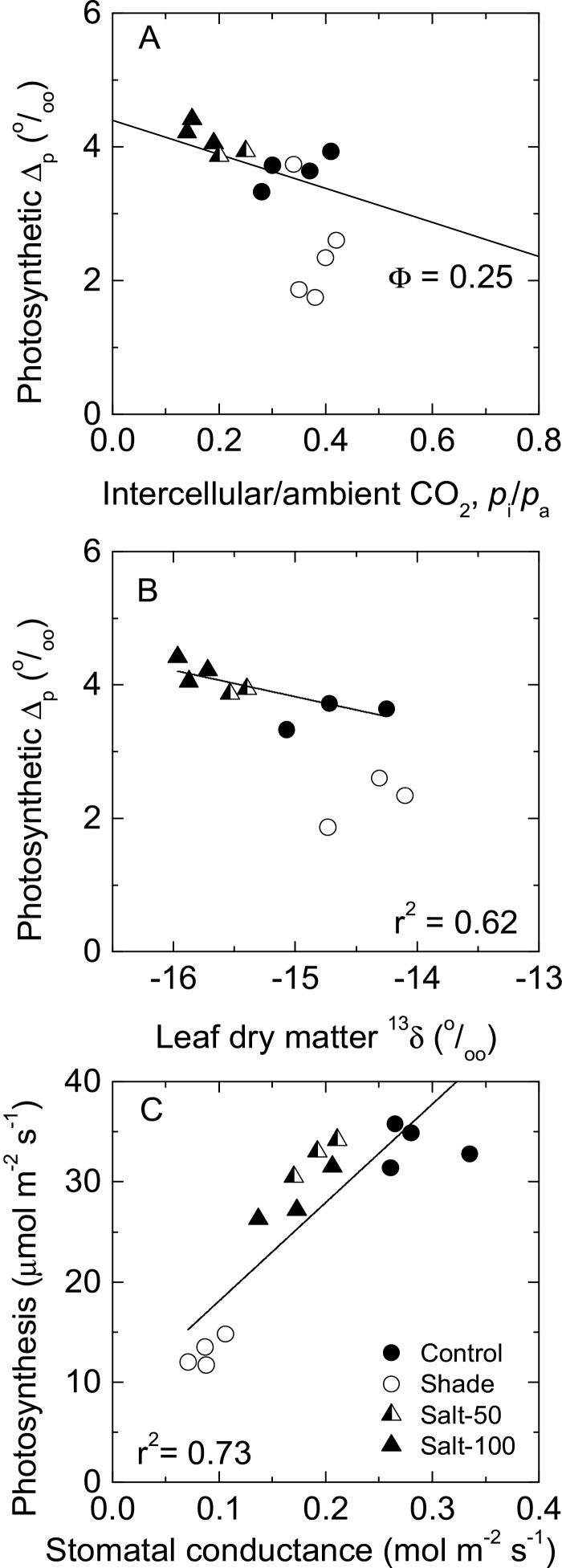
Leaf gas exchange and carbon isotope discrimination in maize plants exposed to salinity and shade. Photosynthetic carbon isotope discrimination, Δ_p_ measured during the gas exchange of maize leaves as a function of intercellular to ambient CO_2_ ratio (A) or leaf dry matter carbon isotope composition, ^13^δ (B). Photosynthetic rates as a function of stomatal conductance are also shown (C). In (A), the solid line is the solution for the C_4_ discrimination model (Farquhar, 1983) using a leakiness (ɸ) value of 0.25. Leaf gas exchange was measured at high light (1800 µmol m^–2^ s^–1^), ambient CO_2_ (400 µl l^–1^) and 26 °C. In (B), the solid line is the linear regression of all data points excluding the shade treatment. In (C), the solid line is the linear regression of all data points. Maize plants were grown in full sunlight and irrigated with water (control, filled circle), 50mM NaCl (Salt-50, half-filled triangle), or 100mM NaCl (Salt-100, filled triangle), or grown in 20% sunlight (shade, open circle).

Leaf water potential (Ψ_L_) decreased in plants exposed to moderate (100mM NaCl) but not mild (50mM NaCl) levels of soil salinity (Table 1). Consequently, photosynthetic rates measured at ambient CO_2_ (*A*_sat_) decreased in maize plants exposed to the higher salinity treatment only (Fig. 1C, Table 1), whereas *g*_*s*_ decreased in plants exposed to both salinity levels (Fig. 1D, Table 1). Plants exposed to shade underwent larger decreases in photosynthesis and *g*_*s*_ (Fig. 1C, D, Table 1). A common linear relationship related *A*_sat_ to *g*_*s*_ (*r* ^2^=0.73) in all the maize plants regardless of the treatment (Fig. 2C).

**Fig. 3.**
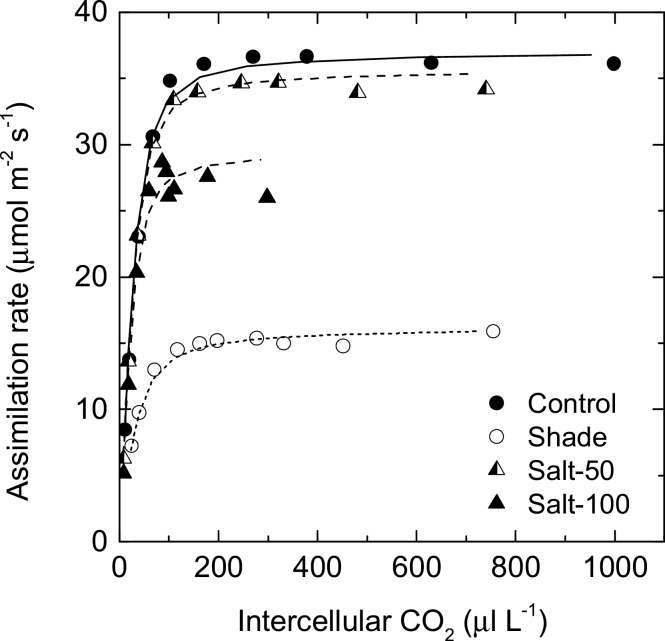
*A*/*C*_i_ response curves for leaves of maize exposed to salinity and shade. Responses of assimilation rates to intercellular CO_2_ (*A*/*C*_i_ curves) measured at a light intensity of 1800 µmol m^–2^ s^–1^ and a leaf temperature of 26 °C. Data points are the average of two replicates. Lines are the mathematical fits using the C_4_ photosynthesis model (von Caemmerer, 2000). Maize plants were grown in full sunlight and irrigated with water (control, filled circle), 50mM NaCl (Salt-50, half-filled triangle), or 100mM NaCl (Salt-100, filled triangle), or grown in 20% sunlight (shade, open circle).

*A*/*C*_i_ curves were fitted using the C_4_ photosynthesis model (von Caemmerer, 2000) to estimate *in vivo V*_cmax_ and *V*_pmax_. Both parameters decreased in the shaded plants relative to the control, while there was a small but non-significant reduction in *V*_cmax_ in the higher salinity treatment. The ratio *V*_pmax_/*V*_cmax_ (2.3–2.8) was similar for all the maize plants, regardless of the treatment (Fig. 3, Table 1).

### Photosynthetic and dry-matter carbon isotope discrimination

Concurrent measurements of ^13^CO_2_/^12^CO_2_ discrimination and leaf gas exchange showed that photosynthetic discrimination (Δ_p_) varied linearly with *p*_i_/*p*_a_ for plants in the control and salinity treatments, yielding a common bundle-sheath ɸ value of 0.25 according to the carbon discrimination model for C_4_ plants (Farquhar, 1983). Thus, salinity changed *p*_i_/*p*_a_ without affecting ɸ. In contrast, shaded plants had lower Δ_p_, *p*_i_/*p*_a_, and ɸ relative to both control and salt-stressed plants (Fig. 2A).

Leaf dry-matter carbon isotope composition (^13^δ) decreased (more negative) significantly in the salt-treated plants only, while shade plants had similar leaf ^13^δ to the control plants (Table 1). Photosynthetic Δ_p_ and leaf dry-matter ^13^δ changed proportionately for the control and salt-treated plants (Fig. 2B). In contrast, the shade plants fell outside the common relationship because their photosynthetic Δ_p_ decreased but not their leaf ^13^δ relative to the control plants (Fig. 2B).

### Activity of photosynthetic enzymes

**Fig. 4.**
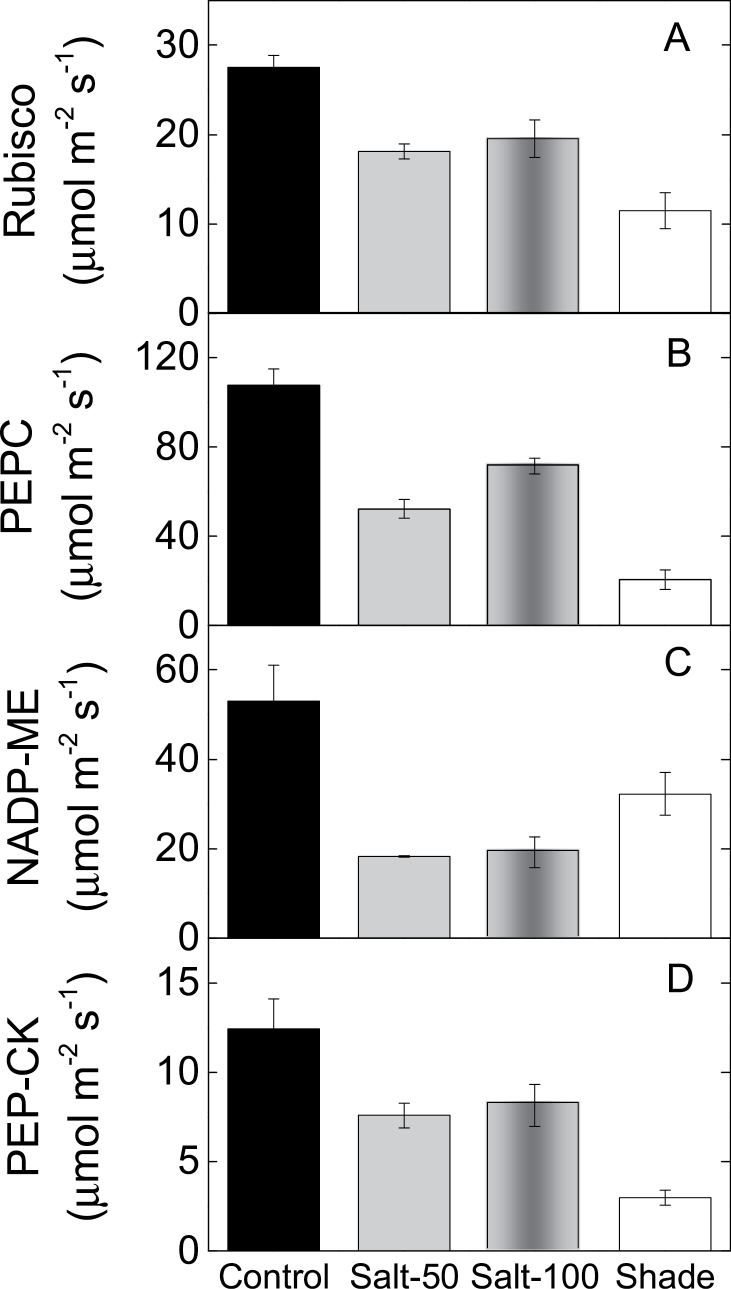
Activity of carboxylases and decarboxylases in maize plants exposed to salinity and shade. Activity of Rubisco (A), PEPC (B), NADP-ME (C), and PEP-CK (D) for maize plants grown in full sunlight and irrigated with water (control), 50mM NaCl (Salt-50), or 100mM NaCl (Salt-100), or grown in 20% sunlight (shade).

**Fig. 5.**
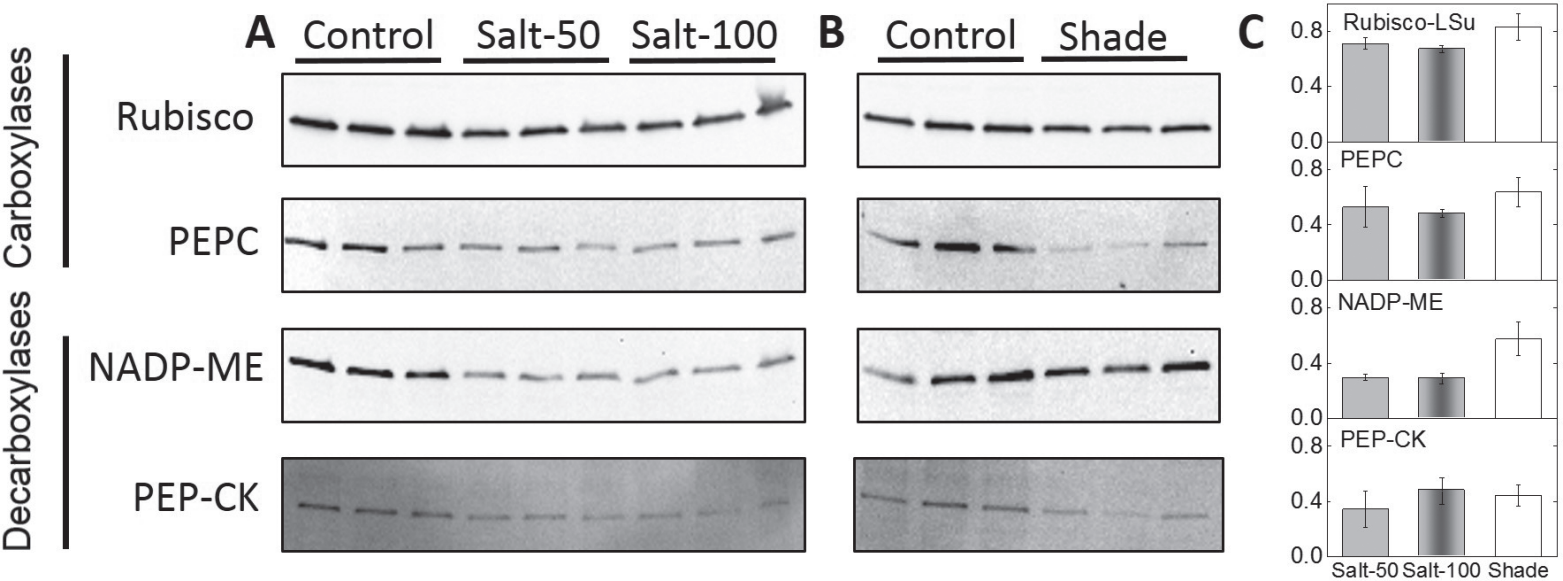
Immunoblots of carboxylases and decarboxylases in maize plants exposed to salinity and shade. Immunoblots of total leaf proteins probed with antisera raised against the four photosynthetic enzymes Rubisco large subunit, PEPC, NADP-ME, and PEP-CK, as described in Materials and methods. The analysis was undertaken separately for the salinity-treated (A) and shade-treated (B) maize plants. Changes in immunoblot densitometry were calculated relative to the control treatment (C).

Leaf Rubisco content and Rubisco activity calculated from *k*_cat_ and the irreversible binding of the transition state analogue [^14^C]CABP decreased by 25 and 50% in the salt-treated and shaded plants, respectively. As expected for C_4_ leaves, Rubisco activity was equivalent to *A*_sat_ for the control and shade leaves; this was not the case for the salt-treated leaves (Fig. 4A, Table 1). Leaf soluble proteins changed together with Rubisco such that Rubisco constituted a constant fraction of soluble proteins under all treatments (Table 1). PEPC activity measured in leaf extracts was reduced by 80% in the shaded plants and by 30–50% in the salt-treated plants relative to the control treatment (Fig. 4B, Table 1). Generally, changes in Rubisco and PEPC activities were reflected by the immunoblots probed with antibodies raised against each of the carboxylase enzymes (Fig. 5). Shading reduced PEPC activity to a greater extent than Rubisco activity, and consequently halved the PEPC/Rubisco activity ratio relative to the control treatment. The PEPC/Rubisco ratio was not significantly affected by salinity in the maize plants (Table 1). It is worth noting that *in vivo* and *in vitro* estimates of Rubisco (*V*_cmax_) and PEPC (*V*_pmax_) activities did not closely correlate in this study. Reconciling these parameters requires more detailed parameterization of C_4_ photosynthesis model (von Caemmerer, 2000).

The activity of the primary decarboxylase NADP-ME and its relative content determined by immunoblot analysis showed 35% reductions across both salinity treatments. NADP-ME activity declined by 60% in the shaded relative to the control plants (Figs 4C and 5, Table 1). Activity and protein expression of the secondary decarboxylase, PEP-CK, was detected in the leaf extracts of all maize plants (Figs 4D and 5). In the control treatment, PEP-CK accounted for 20% of the total C_4_ acid decarboxylation activity measured in maize leaves. This proportion increased to 30% in salt-treated plants and declined to 10% in the shaded plants (Table 1). In absolute terms, PEP-CK activity decreased by 40% in salt-treated plants and by 75% in shaded plants (Figs 4D and 5, Table 1).

## Discussion

### Contrasting impacts of salinity and shade on maize

The main objective of this study was to investigate the regulation of C_4_ photosynthesis subjected to environmental manipulations that are known to have contrasting impacts on the processes of CO_2_ assimilation and diffusion. One of the main acclimation responses to shade is the downregulation of leaf photosynthetic capacity (Boardman, 1977). In contrast, mild to moderate salinity will primarily reduce stomatal conductance by negatively impacting on soil, and hence on leaf water potential. Compared with water stress, salinity has the added advantage of providing a steady stress while avoiding the complications associated with controlling soil water supply (Neumann *et al.*, 1988; Chaves *et al.*, 2009). To this end, both treatments used in this study achieved their goals. While shade markedly reduced plant growth and photosynthetic capacity, salinity reduced stomatal conductance with small effects on photosynthetic rates of the maize plants. Salinity inhibited plant growth to a lesser extent than shade (Table 1).

The evolution of a CCM in higher plants represents a key step to improving photosynthesis under environmental conditions favouring photorespiration by circumventing the inefficiency of Rubisco. The efficient operation of C_4_ photosynthesis requires close coordination between the C_4_ and C_3_ cycles, which is achieved through the distinct cellular compartmentalization of the initial and final carboxylases PEPC (in MCs) and Rubisco (in BSCs), respectively, as well as the localization of the decarboxylases (NADP-ME and PEP-CK for maize) within the BSC. In addition, the maintenance of a high PEPC/Rubisco activity ratio is critical for the build-up of CO_2_ within the BSCs. Importantly, regulating the balance between Rubisco, PEPC, NADP-ME, and other enzymes of the C_3_ and C_4_ cycles allows the dynamic regulation of C_4_ efficiency that other features such as BSC wall conductance or CO_2_ diffusion path length cannot offer in the short to medium term (Hatch, 1987; von Caemmerer and Furbank, 2003). Perturbation of the PEPC/Rubisco ratio by genetically suppressing PEPC results in C_4_ plants unable to grow effectively in air (Dever *et al.*, 1997; Cousins *et al.*, 2007). Leakiness of CO_2_ from the BSCs as determined from measurements of ^13^C/^12^C carbon isotope discrimination represents a key surrogate indicator of the coordination between the C_3_ and C_4_ cycles (Farquhar, 1983). Combining measurements of leakiness with activities of the key enzymes in the C_3_ and C_4_ cycles can elucidate the regulation and efficiency of C_4_ photosynthesis under different environments (Evans *et al.*, 1986; Henderson *et al.*, 1992). Below, we demonstrate that shade, but not salinity, can perturb CCM efficiency as evidenced by changed leakiness.

### Mild to moderate salinity impacts on carbon isotope discrimination through stomatal conductance without affecting leakiness

In maize, mild salinity (50mM NaCl) reduced leaf *g*_s_ but not *A*_sat_, while moderate salinity (100mM NaCl) reduced both *g*_s_ and *A*_sat_. Hence, reduced photosynthetic rates were largely caused by increased resistance to CO_2_ diffusion under both salinity treatments, and this was born out in the lower *p*_i_/*p*_a_ ratio and more negative dry-matter ^13^δ observed in the leaves of salt-treated maize plants (Fig. 2). Reduced stomatal conductance and leaf ^13^δ in response to salinity is commonly reported in C_3_ (Seemann and Critchley, 1985; Brugnoli and Lauteri, 1991) and C_4_ (Bowman *et al.*, 1989; Meinzer *et al.*, 1994; Meinzer and Zhu, 1999) plants. In maize, reduced photosynthetic rates, especially at the highest salinity treatment was also caused by the lower Rubisco and PEPC activities. This reduction was observed in the spectrophotometric assays and the immunodetection of the expressed proteins. Reduced expression of Rubisco under salinity was part of a general reduction in soluble proteins and leaf N. Leaf N is known to decline under salinity due to Cl^–^ interference with nitrate uptake by roots (Munns and Termaat, 1986).

The activity of both carboxylases declined to the same extent under salinity conditions such that the PEPC/Rubisco ratio was indistinguishable from that of the control leaves. This may explain why leakiness was unaffected by salinity in maize leaves despite the changes in photosynthetic Δ_p_ and leaf ^13^δ, which were caused by reduced *p*_i_/*p*_a_ (Fig. 2). In line with these results, when the C_4_ shrub *Atriplex lentiformis* was exposed for 4 weeks to salinity levels equivalent to those used in the current study, photosynthesis and stomatal conductance decreased, while the PEPC/Rubisco ratio remained unchanged until the salinity increased above 120mM. The same study also reported that leakiness, estimated from leaf ^13^δ rather than from photosynthetic Δ_p_, correlated positively with the PEPC/Rubisco ratio (Meinzer and Zhu, 1999). Similarly to *Atriplex*, salinity reduced photosynthesis and increased *p*_i_/*p*_a_ and ɸ values in sugarcane genotypes. Changes in ɸ derived from leaf ^13^δ were also related to the PEPC/Rubisco ratio in sugarcane (Meinzer *et al.*, 1994).

The discrepancy between the studies using *Atriplex* and sugarcane with the current study using maize may be related to a number of factors, the main ones being the salinity level and the basis for leakiness calculation. Meinzer and Zhu (1999) found that mild salinity mainly affected *g*_s_ and had little impacts on ɸ (a similar scenario to the current maize study), and that ɸ and the PEPC/Rubisco ratio were affected at high salinity, indicating profound damage of the photosynthetic apparatus by the accumulating salt, unlike the treatments used in the current maize study. In addition, the difference between leaf ^13^δ and photosynthetic Δ_p_ have not been reconciled yet for C_4_ plants. Post-photosynthetic fractionation of ^13^C/^12^C may be important in C_4_ leaves, thus representing a source of uncertainty in leakiness calculations based on leaf ^13^δ (Henderson *et al.*, 1992).

In maize, both salinity treatments reduced the activity of the primary (NADP-ME) and secondary (PEP-CK) decarboxylases. These observations, together with reduced PEPC activity, suggest that the CCM was down regulated in response to salinity. Results obtained with enzyme activity and immunoblot analysis indicated that the decarboxylases were inhibited more than the carboxylases under salinity. Evidence from transgenic *Flaveria* plants with reduced amounts of NADP-ME have indicated that this decarboxylase is in excess, as photosynthesis was not impacted until activity was reduced to less than 40% of that of wild type (Pengelly *et al.*, 2012). In summary, salinity treatments reduced photosynthesis primarily by reducing *g*_s_ and secondarily by reducing Rubisco and PEPC activities. The balance between the C_3_ and C_4_ cycles was unaffected, as indicated by a similar leakiness between the salt-treated and control maize plants.

### Shade profoundly reduces photosynthetic capacity and leakiness, thus perturbing the coordination between the C_3_ and C_4_ cycles

The shade treatment used in this study had profound impacts on the growth and photosynthesis of the maize plants (Table 1). In particular, shade reduced the photosynthetic capacity measured in terms of *in vivo V*_cmax_ and *V*_pmax_ estimated from the *A*/*C*_i_ curves and in terms of enzyme activity of the carboxylases and decarboxylases. In contrast to salinity, shade had two significantly distinct effects on leaf photosynthesis. Firstly, decreased photosynthetic capacity was mediated by a general downregulation of the activity and protein expression of all measured photosynthetic enzymes. Secondly, the PEPC/Rubisco ratio, photosynthetic Δ_p_, and its derived leakiness decreased relative to those of the control plants, while leaf ^13^δ was not significantly affected (Fig. 2).

The responses of C_4_ photosynthesis to low light vary depending on whether the condition is transient or a short-term acclimation. Under low light (<200 µmol quanta m^–2^ s^–1^), ɸ may increase, possibly as a result of decreased Rubisco activation or increased Rubisco oxygenation due to the low BSC CO_2_ concentration. These factors decrease CO_2_ fixation by Rubisco more than by PEPC, thus maintaining a higher supply of CO_2_ to the BSCs than Rubisco can fix (Henderson *et al.*, 1992; Kromdijk *et al.*, 2008, 2010; Tazoe *et al.*, 2008). However, ɸ in maize leaves was unaffected under conditions of short-term acclimation to low light (Bellasio and Griffiths, 2013*a*, *b*).

In contrast to these studies, leakiness decreased in our study as a result of reduced Δ_p_ with little impact on *p*_i_/*p*_a_, suggesting two main conclusions. Firstly, reduced leakiness in our maize study was accompanied by a reduced PEPC/Rubisco ratio, highlighting the role of this ratio in particular, and the balance between the activity of the C_3_ and C_4_ cycle enzymes in general, for optimizing the efficiency of C_4_ photosynthesis. Our results in maize make it clear that acclimation to low light reduced PEPC activity and protein expression to a greater extent than those of Rubisco. High light dependence of PEPC gene expression is well documented in C_4_ plants (Chollet *et al.*, 1996). Secondly, leaf ^13^δ and photosynthetic Δ_p_ in our maize study did not change together under low light, mainly because the former decreased while the latter was only marginally and not significantly affected by shade (Fig. 2). This is in contrast to a large survey of C_4_ grasses, showing that leaf ^13^δ decreased under shade conditions (Buchmann *et al.*, 1996). On the one hand, our results highlight the problems of using leaf ^13^δ as a proxy for photosynthetic Δ_p_, especially when inferring leakiness and C_4_ regulation. On the other hand, our results point to a stronger dependence of leaf ^13^δ on the diffusive components (salinity effects) within the Δ_p_ equation as opposed to the metabolic factors for which light can have complex effects (Farquhar, 1983; Henderson *et al.*, 1992; von Caemmerer *et al.*, 1997*a*; Ubierna *et al.*, 2011). Solving the link between leaf ^13^δ and photosynthetic Δ_p_ remains a key challenge for elucidating the underpinnings of carbon isotope discrimination in C_4_ leaves.

In another contrast with the salinity treatments, shade reduced the activity of the primary decarboxylase NADP-ME less, while strongly suppressing the activity of the secondary decarboxylase PEP-CK. Taken together, these results constitute rare evidence for decarboxylase flexibility in response to environmental conditions, with salinity and shade having opposite effects on the ratio of PEP-CK to NADP-ME activity in maize. It is unlikely that the observed changes in NADP-ME and PEP-CK were due to anaplerotic activities due to their low contribution relative to that of the photosynthetic isoforms (Drincovich *et al.*, 2001). The differential engagement of the decarboxylation pathways enables C_4_ plants to acclimate to varying conditions of light (Furbank, 2011). For example, it has been shown that the flexible operation of NADP-ME and PEP-CK decarboxylases in maize allows the bundle sheath to regulate NADPH supply under variable light conditions (Bellasio and Griffiths, 2013*b*). In the current study, we demonstrated the differential engagement of the primary and secondary decarboxylases under long-term acclimation to low light through the significant reductions of PEP-CK activity and protein content (Figs 4 and 5).

In summary, we demonstrated that long-term acclimation to low light in maize causes a reduction in BSC leakiness. This reduction was underpinned by a greater downregulation of PEPC activity and content relative to those of Rubisco, and by a flexible partitioning of C_4_ acid decarboxylation activity between NADP-ME and PEP-CK.

## Abbreviations:

BSC: bundle-sheath cell
CABP: carboxyarabinitol bisphosphate
CCM: CO_2_ concentration mechanism
MC: mesophyll cell
NADP-ME: nicotinamide adenine dinucleotide phosphate malic enzyme
PEPC: phosphoenolpyruvate carboxylase
PEP-CK: phosphoenolpyruvate carboxykinase
Rubisco: ribulose-1,5-bisphosphate carboxylase/oxygenase.
